# Implications of Cattle Trade for the Spread and Control of Infectious Diseases in Slovenia

**DOI:** 10.3389/fvets.2019.00454

**Published:** 2020-01-14

**Authors:** Tanja Knific, Matjaž Ocepek, Andrej Kirbiš, Hartmut H. K. Lentz

**Affiliations:** ^1^Veterinary Faculty, Institute of Microbiology and Parasitology, University of Ljubljana, Ljubljana, Slovenia; ^2^Veterinary Faculty, Institute of Food Safety, Feed and Environment, University of Ljubljana, Ljubljana, Slovenia; ^3^Institute of Epidemiology, Friedrich-Loeffler-Institut, Greifswald, Germany

**Keywords:** cattle movements, network analysis, transmission, temporal network, SI model, *Mycobacterium avium* subsp. *paratuberculosis* (MAP)

## Abstract

The objectives of this study were to gain insight into the structure of the cattle trade network in Slovenia and to evaluate the potential for infectious disease spread through movements. The study considered cattle movements between different types of premises that occurred between August 1, 2011 and July 31, 2016 with the exclusion of the movements to the end nodes (e.g., slaughterhouses). In the first part, we performed a static network analysis on monthly and yearly snapshots of the network. These time scales reflect our interest in slowly spreading pathogens; namely *Mycobacterium avium* subsp. *paratuberculosis* (MAP), which causes paratuberculosis, a worldwide economically important disease. The results showed consistency in the network measures over time; nevertheless, it was evident that year to year contacts between premises were changing. The importance of individual premises for the network connectedness was highly heterogeneous and the most influential premises in the network were collection centers, mountain pastures, and pastures. Compared to random node removal, targeted removal informed by ranking based on local network measures from previous years was substantially more effective in network disassociation. Inclusion of the latest movement data improved the results. In the second part, we simulated disease spread using a Susceptible-Infectious (SI) model on the temporal network. The SI model was based on the empirically estimated true prevalence of paratuberculosis in Slovenia and four scenarios for probabilities of transmission. Different probabilities were realized by the generation of new networks with the corresponding proportion of contacts which were randomly selected from the original network. These diluted networks served as substrates for simulation of MAP spread. The probability of transmission had a significant influence on the velocity of disease spread through the network. The peaks in daily incidence rates of infected herds were observed at the end of the grazing period. Our results suggest that network analysis may provide support in the optimization of paratuberculosis surveillance and intervention in Slovenia. The approach of simulating disease spread on a diluted network may also be used to model other transmission pathways between herds.

## Introduction

In cattle, *Mycobacterium avium* subsp. *paratuberculosis* (MAP) causes paratuberculosis, a worldwide endemic disease with large economic consequences (1, 2). Paratuberculosis usually occurs as a subclinical infection, but some animals develop a chronic wasting disorder, which results in decreased milk production and pregnancy rate and increased culling and mortality rate (3, 4). Even though subclinically infected animals appear healthy, they are associated with financial losses (5) and are considered the most important factor contributing to the spread of the disease between herds (6). The fecal-oral transmission is believed to be the most common route of within-herd transmission of MAP. Infection may also be spread with colostrum and milk from infected animals and intrauterine transmission. Additionally, an alternative route of transmission with bioaerosols has been suggested (7). Calves are the most susceptible; nevertheless, infections of adult animals occur more often and may play a more important role in the on-farm persistence of the disease than it was previously expected (8, 9). The progression of the infection follows two different paths: some animals become constant high-shedders and months or years after, the infection manifests clinical signs, while other animals shed MAP in various amounts intermittently or not at all and remain asymptomatic (10). The main route of paratuberculosis spread between cattle herds is believed to be by movements of infected animals (1). Studies on empirical data showed that between-farm transmission of MAP is significantly associated with animal movements. This was shown through coupling the network analysis of livestock movements with genotyping of MAP strains on farms (11) and with the disease statuses of farms (12).

Besides cattle and small ruminants, MAP is able to infect and cause clinical signs in many other domestic (e.g., chicken, dog) and wild animal species (e.g., rabbit, fox, deer, non-human primates) (13). It may also be connected with Crohn's disease and some other human diseases, although the discussion about MAP being a zoonosis is ongoing and controversial (14, 15). Potential sources of human exposure to MAP are contaminated food (e.g., milk and milk products, raw meat), water, environment and direct contact with infected animals (16). Garcia et al. (17) pointed out that in the case of a confirmed link between MAP and Crohn's disease in humans, we can expect significant economic losses that will disproportionally affect the dairy industry.

In Europe, the animal level prevalence of paratuberculosis in cattle was estimated to be ~20%, with the lowest estimates of 3–5% (18). For countries with significant dairy industry, the herd level prevalence is presumed to be a lot higher than 50% (1). Paratuberculosis is also common in ruminants in Slovenia (19). The last prevalence study in Slovenia was conducted in 2008; it was estimated that 18.49% of cattle herds were infected, while the estimated true-prevalence at the animal level was 3.96% (20). Ocepek et al. (21) argued that the low disease prevalence in Slovenia could be partly explained by the existence of numerous small herds since within-herd spread is more probable than between-herd spread. In the last few years, the average size of agricultural holdings in Slovenia increased, thus providing better conditions for the spread of MAP. We presume that the prevalence of paratuberculosis in Slovenia is now higher compared to the estimates from a decade ago. The need for surveillance and control measures for paratuberculosis in Slovenia is evident from two aspects. The first is significant direct disease losses in the cattle industry, which are especially important for the dairy sector since in Slovenia, the milk output accounts for almost a third of the whole livestock outputs (22). In addition, the cattle industry may be subject to indirect disease losses due to possible trade bans. The second is the increasing concern for public health and efforts to assess and subsequently lower the human exposure to pathogens through food of animal origin.

Determination of paratuberculosis prevalence as well as the effectiveness of intervention programs is impeded by the long incubation period, intermittent shedding and inadequate diagnostic tests (6). When assessing the impact of paratuberculosis, its potential for spreading and efficacy of different surveillance and control measures, the problems of the lack of empirical data and scarce resources can be overcome to a certain extent by the use of different mathematical and computational methods. In the case of paratuberculosis, these methods were used to study: the disease dynamics at the herd level (23–25), between-herd transmission and spread potential (11, 26), evaluation of the surveillance systems and control measures (27–29), and assessment of economic consequences of the disease and control measures (30–34).

Among those methods, network analysis of animal movements has proved to be particularly useful for studying the potential spread of disease among premises and a risk-based selection of premises that can serve as targets for surveillance and intervention measures (26, 35–37). Network analysis enables a systematic investigation of animal trade and allows the evaluation of risk potential for disease transmission. Specifically, local network measures or node centrality measures can capture the epidemiological importance of the individual premises in the network. Since hierarchical structure is a typical characteristic of the real world networks, depending on the application centrality measures can be used to detect premises that pose a higher risk for disease introduction or spread or act as bridges between subgroups (38). In such a way informed surveillance and control measures are epidemiologically and economically more effective compared to non-targeted measures (39–41). Although the fact that animal movements play an important role in between-farm disease transmission is well-known, in Slovenia, it is still not exploited for informed national disease surveillance and control programs.

The objectives of this study were to provide an in-depth insight into the cattle trade in Slovenia and assess the potential spread of MAP through cattle movements. To obtain a tangible overview of the cattle trade structure, we described the static yearly and monthly snapshots of the network. These time resolutions are convenient for capturing meaningful characteristics of animal trade and allow for a simple assessment of changes through time. We were especially interested in the consistency and heterogeneity of the local network measures of individual premises to evaluate the potential benefits of network analysis for informed risk-based paratuberculosis surveillance and intervention measures. To better understand the implications of the network structure, we modeled the possible spread of MAP on the temporal network and therefore utilized the unfolding accessibility method that efficiently models the epidemic spreading on temporal networks (42).

## Materials and Methods

### Movement Data

The data on cattle movements from January 1, 2010 to August 5, 2016 were obtained from the Administration of the Republic of Slovenia for Food Safety, Veterinary Sector and Plant Protection of the Ministry of Agriculture, Forestry and Food. The administration holds the data of the life history events of each individual bovine animal including all movements between all types of premises within Slovenia and with import or export countries in the central register of bovine animals. Data provided by the government were stored in two datasets. In the first dataset, each line denoted the movement of an individual animal on a particular date; namely: country of origin, animal ID, source premises, target premises, date of movement, date of birth, sex, breed, type of movement and import or export country. The second dataset included premises ID, municipality, type of premises and geographic coordinates.

We extracted the information on the movements for the last five available years, namely data from August 1, 2011 to July 31, 2016. As we were interested in movements that are epidemiologically important for the spread of disease, we omitted all movements to premises that corresponded to end nodes. These are premises that animals never leave, at least not alive. Therefore, we excluded movements to slaughterhouses, animal waste processing centers, and corpse collection centers. For export movements, the use of animals at the target destination was not included in the dataset, consequently, the export movements to end nodes remained in the data. Included types of premises were farm holdings, pastures, mountain pastures, collection centers, exhibitions, and fairs. Collection centers are registered facilities where animals from different premises are grouped together intended for movements in consignments, whether for transfer, trade, import, or export. Similarly, fairs are also registered facilities, but animals in consignments are intended only for trade (Rules on the conditions to be met by the assembly center operators and retailers with animals, Official Gazette of RS, No. 99/06, 96/09 and 42/10)^1^. Exhibitions are events where animals are usually not grouped together and trade is not the primary aim. At the collection centers, exhibitions, and fairs animals stay only for a day or a couple of days. At the pastures and mountain pastures they usually spend the whole pastoral season. In Slovenia, the pastoral season starts at the beginning of April and lasts until the middle of November.

For the 345 premises with missing information on the type of premises, we performed an individual search in the VOLOS portal, where the central register of bovine animals is accessible. For the remaining 27 premises with an unknown type, we analyzed the cumulative trade volume as described in Koeppel et al. (43) and assigned the type of premises accordingly.

We believe that even though unrecorded movements may have been present, they did not hinder the validity of the represented cattle trade network considerably. In Slovenia, bovine animals must be marked as soon as possible, mandatorily before the first movement, but the latest until the age of 20 days and all movements must be registered (Rules on the identification and registration of bovine animals, Official Gazette of RS, no. 50/16)^2^. In the analysis, we used the national data and based on the proportion of active premises, we assume that the large majority of movements are indeed registered. For example, in 2013 28,284 farm holdings were involved in cattle trade, according to census data this was 82.98% of farm holdings with cattle^3^.

### Network Analysis

The final trade dataset consisted of source and target premises, date of movement and batch size. Only premises involved in animal trade during the 5-year study period were included in the analysis. To get a tangible overview of the cattle trade network structure, we considered two time windows and constructed sixty monthly and five yearly static networks. Networks of premises (nodes) connected through cattle movements (arcs) were generated in the Pajek software (44). In each of these networks, we aggregated individual movements between the same two premises into batches when the direction of movements coincided. Networks were directed (movement is directed to premises receiving the animal) and weighted (the number of moved animals between pairs of premises varied). Nodes had an assigned type of premises. Network analysis was performed using the Pajek software and the igraph package in the R programming language (45). Premises were regarded as active if they participated in at least one animal movement in the specified period. Import and export countries appear as single nodes in the dataset and were thus excluded from the calculation of network measures.

In order for the animal movements to be of appropriate assistance to the informed risk-based targeted surveillance and control measures, the network of movements should exhibit three key characteristics. First, it has to show some consistency of properties over time. Second, premises ought to be of heterogeneous importance to the overall connectivity of the network. Third, associated with the latter, the structure of the network should follow some order. In other words, the structure should not have emerged only by chance. We explored the characteristics of cattle movement networks through global measures, namely by the number of active premises, sizes of giant weakly and strongly connected components (GWCC and GSCC, respectively), bow-tie partition, density, average shortest path length, diameter, average degree, clustering coefficient, and degree assortativity (46, 47). In local network measures or node centrality measures, we focused on measures that were recognized as important for disease transmission in previously published studies (36, 37, 39, 40, 48–52). We calculated the unweighted and weighted total, in- and out-degree, betweenness centrality and total, in- and out-degree closeness centrality (46). We evaluated the consistency of global and local network measures at monthly and yearly resolutions. Using a Mann–Kendall test, we investigated whether there was any trend in the network measures on a monthly resolution.

Correlations of node level measures across years and between different measures were assessed with Spearman's rank correlation coefficients (36, 37, 48, 52). We assessed the heterogeneity of premises contributions to the overall network connectivity utilizing degree distributions (46). Since the degree distributions are heavy-tailed, we plotted yearly and monthly distributions using a logarithmic scale. The number of contacts contributed by nodes with highest degrees was calculated. We tested for the differences in centrality measures between different types of premises using Kruskal–Wallis rank sum test and *post-hoc* multiple comparison test (36, 48). To test whether the features of the Slovenian cattle movement networks could have occurred simply by chance, we compared the average path length, diameter, clustering coefficient, assortativity, size of GWCC and GSCC of our yearly networks to 1,000 random networks (Erdős–Rényi networks) of the same size and density (36, 37, 40).

Since local network measures give information on the relative importance of each node in the network, we can use them to simulate targeted intervention measures. In network analysis, this means removing nodes with the highest centralities from the network (37, 39, 53). In practice, this could be implemented as targeted surveillance, movement restriction, isolation, culling, vaccination or increased biosecurity measures. Due to the fact that decisions on future intervention measures must be made based on the currently available data, values from previous years were used to rank nodes. Nodes were sorted in decreasing order by a pre-calculated selected measure and then removed one by one. We simulated targeted intervention measures by removing 400 nodes from the fifth yearly network based on total degree, betweenness and closeness centrality from the same year and from previous years. At each step, the size of the GSCC for the remaining network was calculated, since the behavior of the GSCC mimics the functionality of the network. In addition, we simulated the removal of nodes by random ordering.

### Inclusion of Time Data for Disease Spread

So far, the considered system was treated as static, which means that the temporal nature of cattle movements was neglected. Since all cattle movements are provided with timestamps, it is possible to simulate a potential disease spread on the network explicitly over time. This circumstance cannot be addressed using standard network analysis; therefore, temporal network analysis has emerged over the last years (38, 53). A static network can be represented as a list of arcs *e*, where each element contains a contact between nodes. As an example, the list of arcs *e* = [(1, 2), (3, 4)] would represent a network, where there is an arc from node one to node two and an arc from node three to node four. In *temporal networks*, trade contacts are time-stamped and are represented as a list of triples, e.g., *e_temporal* = [(1, 2, 1), (3, 4, 2), (1, 2, 3)] means that the contact from node one to node two was active at times one and three and the contact from node three to node four was active at time two. In order to distinguish contacts between nodes in temporal networks from static ones, we call a temporal contact from node i to j at time t (i, j, t) an *edge*, whereas there is an *arc* (i, j) between i and j in the static network, when there was at least one edge in the studied period.

To assess the possible spread of MAP through cattle movements in the studied period, we simulated the spread of disease using a Susceptible-Infectious (SI) model using the temporal trade data explicitly. The analysis was carried out using the Python programming language (54). We adopted the model developed by Lentz et al. (42). The original SI model was developed to take into account causality by considering the succession of movements and thus it provides the so-called unfolding of the accessibility graph of a temporal network. This is also known as network reachability or out-going contact chain (51). Compared to other methods, the method used here computes the out-going infection chain of all nodes at the same time efficiently. It thus generates the whole reachability matrix in a single run. The original model describes a possible spread of disease if the probability of transmission is equal to one. For a detailed description of the model, see Lentz et al. (42, 53) and the developed Python code^4^.

**Figure 1:**
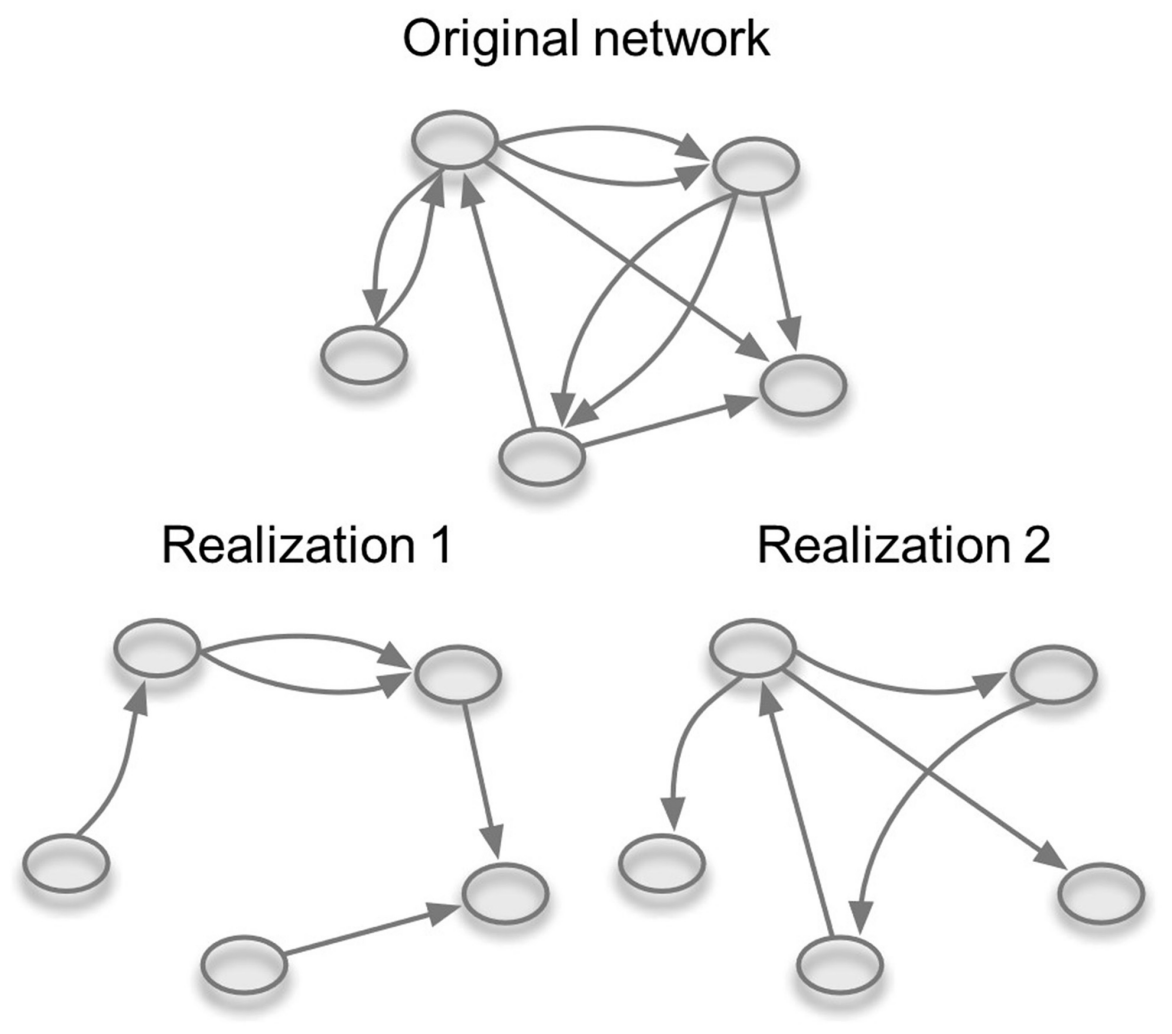
Dilution of the temporal network. Based on the value of the probability of transmission (p) random edges are omitted from the network. Two realizations of the same network for p = 0.5 are shown.

In order to simulate the spread of MAP on our studied network, the possibility of applying a probability of transmission with values <1 had to be implemented. Therefore, we improved the model of Lentz et al. (53). Different transmission probabilities were realized with a dilution of the initial studied temporal network (Figure 1). This means that the probability of infection per movement was modeled by removing a certain number of edges from the whole network. Statistically speaking we replaced the probability of transmission by another probability, namely the probability of contact. More precisely, if p = β × τ is the probability of transmission between two nodes (where β is the infection probability given contact and τ is the contact probability), we set β to one and vary τ in order to adjust p. The dilution of the network was carried out by a random sampling of edges from the whole temporal network based on the value of the transmission probability we wanted to simulate. To be specific, the proportion of edges in the diluted network was equal to the chosen probability of transmission. Since the edges were kept in the diluted network based on a random sampling and a deterministic value for the probability of transmission was used, we adopted two limiting assumptions underlying the implemented SI model. First, a within-herd prevalence is assumed to be the same in all infected herds and second, when an infected animal is moved, the receiving herd always becomes infected. Therefore, in this case, the probability of transmission can also be described as the within-herd prevalence or the probability that the moved animal is diseased. This model can be used to study the between farm spread of any disease that primarily spreads with animal movements. Here, we used the initial conditions and parameter values corresponding to the latest available data on paratuberculosis within-herd and herd prevalence in Slovenia.

We used the temporal network with the full available time resolution (1 day) as a substrate for paratuberculosis spread, and we tracked the spread between premises for 1,826 days. We evaluated the influence on paratuberculosis spread of four different values for the probability of transmission (p). The estimated scenario (p = 0.25) reflects the results of a study of within-herd prevalence conducted in 2010, where they estimated prevalence in three different herds in Slovenia based on four combinations of three diagnostic methods and three different samples (4). Since the study included only three herds, significant uncertainty is associated with the estimate. For this reason, additional scenarios were included based on estimations of the within-herd prevalence from the literature; namely: best-case scenario (p = 0.02), middle scenario (p = 0.15), and worst-case scenario (p = 0.40) (26). We prepared one hundred diluted networks for each scenario.

Next, we selected the initially infected premises by random sampling among all active farm holdings in the studied period. In 2008, the true prevalence of paratuberculosis in Slovenia was estimated at 18.49% (20); accordingly, we selected 6,818 farm holdings as initially infected. For each diluted network, we performed ten simulations of the SI model with randomly selected initially infected farms according to the estimated prevalence. Even though we sampled only from farm holdings, we kept all types of premises in the network. Consequently, we allowed for the transmission of disease, even if a pair of premises was not directly connected, but rather connected via a mountain pasture or a fair for instance.

Using the above-mentioned network dilution and initial conditions, we simulated the SI-infection spreading for 100 diluted networks and ten realizations of initial conditions for each probability of transmission, yielding 1,000 simulations per transmission probability. The output of the model was the number of infected farm holdings at each time step, i.e., 1,000 infection curves for each transmission probability. In addition, we calculated the slopes of all infection curves in order to highlight significant incidence changes.

## Results

### Description of Cattle Movements

Out of 2,277,166 recorded individual animal movements from August 1, 2011 to July 31, 2016, 1,020,860 met the inclusion criteria (Table 1). Movements within Slovenia represented 71.3%, the rest were animals imported and exported from or to EU and non-EU countries. For imported animals, only the ones intended for further breading were accounted for, whereas for the export, animals moved to end nodes were also included. Within the country, 543,180 animals were moved from one to 28 times, the majority of them (97.4%) being moved up to three times. Collectively, 37,138 premises were involved in cattle trade; namely: 36,885 farm holdings, 36 pastures, 174 mountain pastures, 16 exhibitions, 3 fairs, and 24 collection centers. Not all premises were active during the entire study period, several have ceased activities and few premises were newly established. This is also evident from the national statistical data since the number of farm holdings with cattle was decreasing through time. In 2016, there were 32,805 farm holdings with cattle registered in Slovenia, which was 3,314 less than in 2010^5^.

**Table 1:** Summary of cattle movement data included in the study.

**Study year**	**Year 1**	**Year 2**	**Year 3**	**Year 4**	**Year 5**	**Total**
**Study period**	**Aug 2011–Jul 2012**	**Aug 2012–Jul 2013**	**Aug 2013–Jul 2014**	**Aug 2014–Jul 2015**	**Aug 2015–Jul 2016**	**Aug 2011–Jul 2016**
No. of active premises	29,172	29,004	28,395	28,195	28,008	37,138
No. of animal movements	142,142	144,589	144,050	144,834	151,992	727,607
No. of imported cattle for further breading	20,510	18,650	20,281	23,673	25,419	108,533
No. of exported cattle	38,073	40,970	39,184	30,748	35,745	184,720

**Figure 2:**
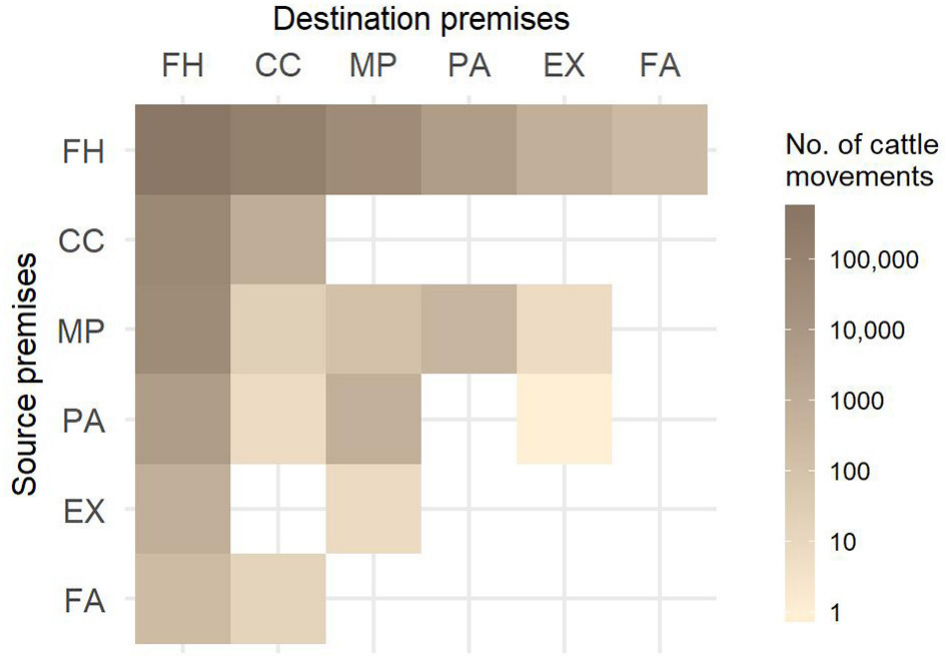
Number of animal movements between different types of premises from August 1, 2011 to July 31, 2016. FH, farm holdings; CC, collection centers; MP, mountain pastures; PA, pastures; EX, exhibitions; FA, fairs.

**Figure 3:**
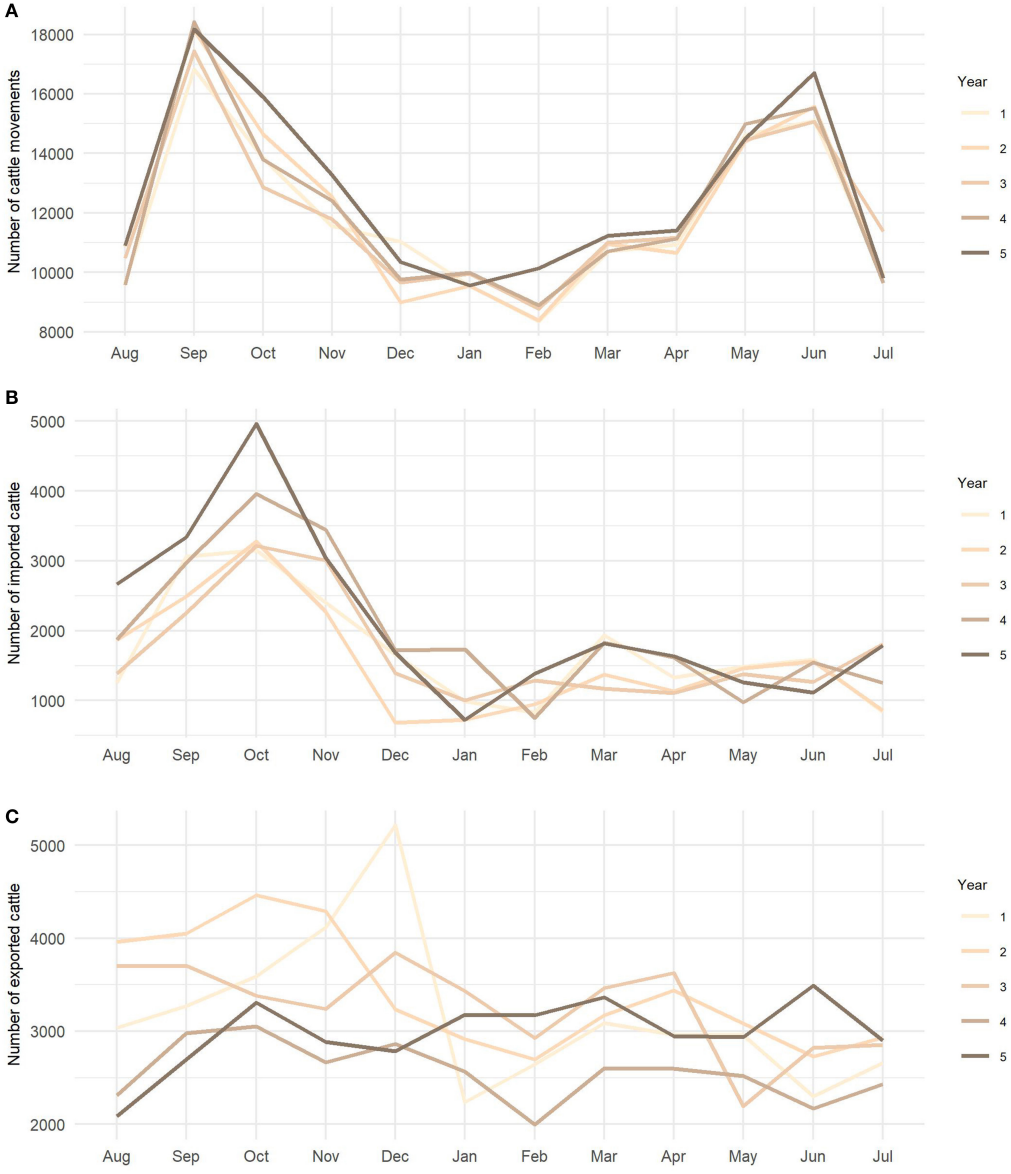
Number of animal movements by month from August 1, 2011 to July 31, 2016. **(A)** Number of cattle movements in Slovenia without movements to end nodes, **(B)** number of cattle imported for further breading, and **(C)** number of exported cattle including movements to end nodes.

More than half of all movements in Slovenia created direct farm-to-farm contacts and almost a third (29.3%) went through collection centers (Figure 2). The number of movements by month revealed a strong seasonal pattern, each year with the maximum number of movements in September and another peak in June. The minimum number of movements was in February, except for the last studied year when January was the least active month (Figure 3). The number of imported cattle intended for further breeding by month also showed consistency throughout all five years with peak activity in autumn. Most of these animals were imported from the Czech Republic, Hungary, Romania, Slovakia and Austria (58.2, 10.2, 9.5, 9.2, and 3.4%, respectively). Overall, approximately half of the animals were imported directly to farm holdings and half went through collection centers. The proportion of animals going through collection centers increased every year; from 41% in the first year to 61% in the last studied year. The export movements did not show pronounced consistency or seasonality. In the last studied year, Slovenia exported 35,745 animals. Overall, most animals (88.9%) were exported to Austria, Italy, and Lebanon.

### Characterization of the Cattle Trade Network

Global network measures for monthly and yearly networks are summarized in Table 2. The total number of active nodes in yearly networks decreased through the years. Based on node types we saw that only the number of active farm holdings dropped each year, from 78.5 to 75.3% in the last year, whereas the number of active nodes per other types did not change considerably. Each year, there were 20 active collection centers. There were one to a maximum of two fairs per year and two to seven exhibitions. The number of active pastures and mountain pastures varied a little; in the first year, there were 26 active pastures and 32 in the last year, while the number of active mountain pastures was 170 in the first year and 162 in the last year. The minimum number of arcs was noted in the first year and the maximum number in the last studied year. This does not apply for the number of batches since the minimum number of batches was recorded in the last year. In monthly networks, the number of active farm holdings, pastures, and mountain pastures showed seasonal patterns, whereas the number of active collection centers, fairs and exhibitions varied very little between months. The number of arcs varied considerably between months, but very little in the same month in different years.

**Table 2:** Summary of global measures of yearly and monthly cattle movement networks.

	**Yearly networks Mean [range]**		**Monthly networks Mean [range]**	
Number of active premises	28,554.80	[28,008–29,172]	7,926	[6,782–9,128]
Number of movements	145,521.40	[142,142–151,992]	12,127	[8,364–18,415]
Number of batches	60,120.40	[59,310–61,262]	6,665	[5,448–7,817]
Density	4.36 × 10^−5^	[4.30 × 10^−5^−4.44 × 10^−5^]	4.83 × 10^−6^	[3.95 × 10^−6^−5.67 × 10^−6^]
Average degree	3.24	[3.19–3.30]	0.36	[0.29–0.42]
Average shortest path length	9.34	[8.85–10.16]	2.71	[1.72–5.52]
Diameter	28.6	[25–37]	9.25	[7–16]
Degree assortativity	−0.0645	[−0.0653 to −0.063]	−0.0689	[−0.157 to −0.047]
Clustering coefficient	0.0017	[0.0016–0.0018]	7.47 × 10^−4^	[4.41 × 10^−4^−1.43 × 10^−3^]
Size of GWCC	27,828	[27,331–28,416]	3,644.70	[2627–4960]
% of active holdings	97.46	[97.15–97.82]	45.82	[36.88–56.48]
Size of GSCC	5,185.60	[4,791–5,455]	12.92	[3–73]
% of active holdings	18.17	[16.42–19.48]	0.16	[0.04–0.93]

*Given numbers are mean values over time*.

*GWCC, giant weakly connected component; GSCC, giant strongly connected component*.

The arc density shows that both yearly and monthly networks are sparse since on average only 4.36 × 10^−3^ and 4.83 × 10^−4^% of contacts are realized, respectively. In the yearly networks, the total degree of a single node ranged between zero and 1,757, with the mode being one and median two. The total degree in a monthly network ranged between zero and 364, with mode and median being zero. The average shortest path length showed that on average approximately nine animal movements are connecting a pair of randomly chosen active premises in the yearly networks and three animal movements in monthly networks. The average network diameter or the longest shortest path between two active nodes in a network required ~29 steps in the yearly and nine in the monthly networks. All networks had negative degree assortativity, which means that a connection between nodes with different degrees is more likely than a connection between nodes with a similar degree. Small values of the clustering coefficients showed that the clustering in the analyzed networks was weak. A Mann–Kendall test did not show any trend in the monthly network measures.

In each of the studied yearly networks, almost all active nodes were part of GWCC, whereas less than half of all active nodes in the monthly networks were also part of the GWCC. The yearly GWCC on average included 75% of all nodes (range: 73.6–76.5%). In monthly networks, the proportion of nodes in the GWCC was lower, on average 9.81% and ranged between 7.1 and 13.4%. The sizes of the second largest weakly connected components were significantly smaller and included from 6 to 9 nodes in yearly networks and from 17 to 120 nodes in monthly networks. In the yearly networks, the GSCCs on average contained 14% of all nodes, with a minimum number of nodes in the first year and a maximum in the last studied year. The second largest strongly connected component included from seven to 21 nodes with the average being 13.4 nodes. There were one to four strongly connected components with more than ten nodes per year. The GSCCs in each monthly network contained <1% of active nodes. GSCCs with more than 20 nodes were observed in June, July, August, and October. The second largest strongly connected component on average consisted of six nodes (range: 3–16 nodes). In the large majority of months, only one strongly connected component included more than ten nodes.

**Figure 4:**
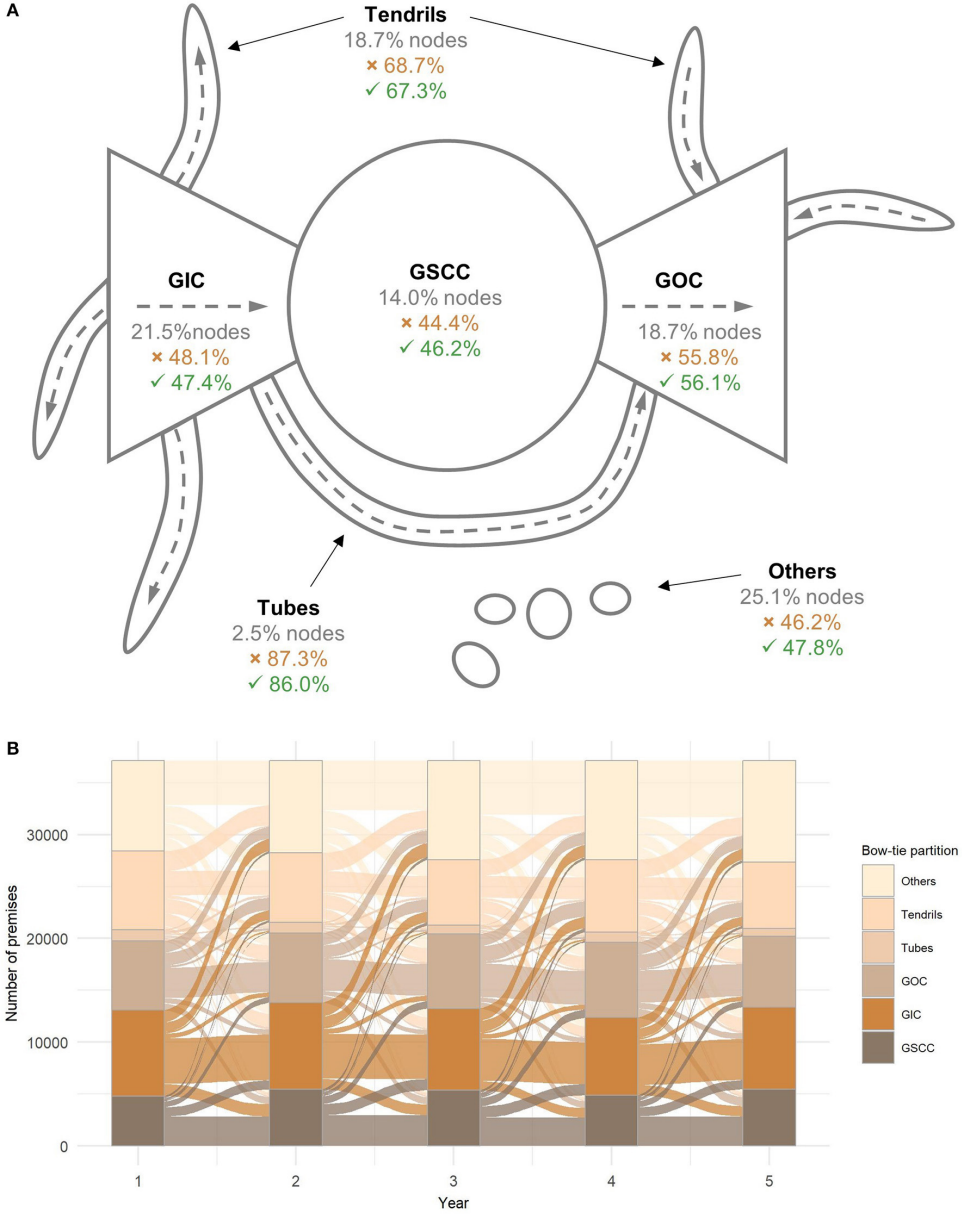
Bow-tie partition. **(A)** Structure of the bow-tie partition of the yearly cattle movement networks. Gray numbers are the average percentage of nodes in a component. The percentage of nodes exiting the component is denoted with (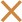) and entering the component is denoted with (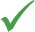) (55). **(B)** Transition plot showing the sizes of components per year and the transition between bow-tie components. GSCC, giant strongly connected component; GIC, giant in component; GOC, giant out component.

From the epidemiological perspective, the component structure is an important feature of networks since it determines possible paths for the transmission of disease and the maximum size of disease outbreaks. A so-called bow-tie partition assigns nodes into six different components; namely GSCC, *giant in component* (GIC), *giant out component* (GOC), *tubes, tendrils*, and *others* (55). The size of the GWCC equals all the nodes in the network minus nodes in the component *others*. The situation is most unfavorable if an outbreak starts in nodes that belong to the GSCC or GIC because there are many available paths for the spread of disease. On the contrary, a disease originating in nodes from other components can reach only a limited number of nodes (53). Figure 4 displays the structure of the bow-tie partition of the average yearly network in the studied period and the mean percentage of nodes belonging to each component. The affiliation of nodes to different bow-tie components is not constant over the years. In fact, more than half of the nodes changed their affiliation each year, which shows that even though the node level measures were highly correlated, the overall connectedness of the premises to other parts of the network tended to change over time (Figure 4).

Local network measures showed considerable consistency across the studied period. Correlations of all measures between years were moderate to strong and highly statistically significant (adjusted *p*-values < 0.0001). For all measures, the minimum Spearman's rank correlation coefficients were found when comparing first and last studied year (Spearman's ρ = 0.39–0.61), whereas all except one maximum coefficient were found between the fourth and fifth year (Spearman's ρ = 0.52–0.72). An exception was the correlation coefficient for out-closeness centrality, for which the maximum value was found between the third and fourth year.

Correlations between local network measures were also highly statistically significant (adjusted *p*-values < 0.0001). Degree, weighted degree, betweenness, and closeness centralities were moderate to very strongly correlated with all other network measures (Spearman's ρ = 0.44–0.94). Centralities based on in-going movements in-degree, in-weighted degree, and in-closeness centrality were very strongly correlated between each other (Spearman's ρ = 0.91–0.97). The same applies to centralities concerning out-going movements. Very week correlation was found only when comparing in-going and out-going centralities (Spearman's ρ = 0.08–0.16).

**Figure 5:**
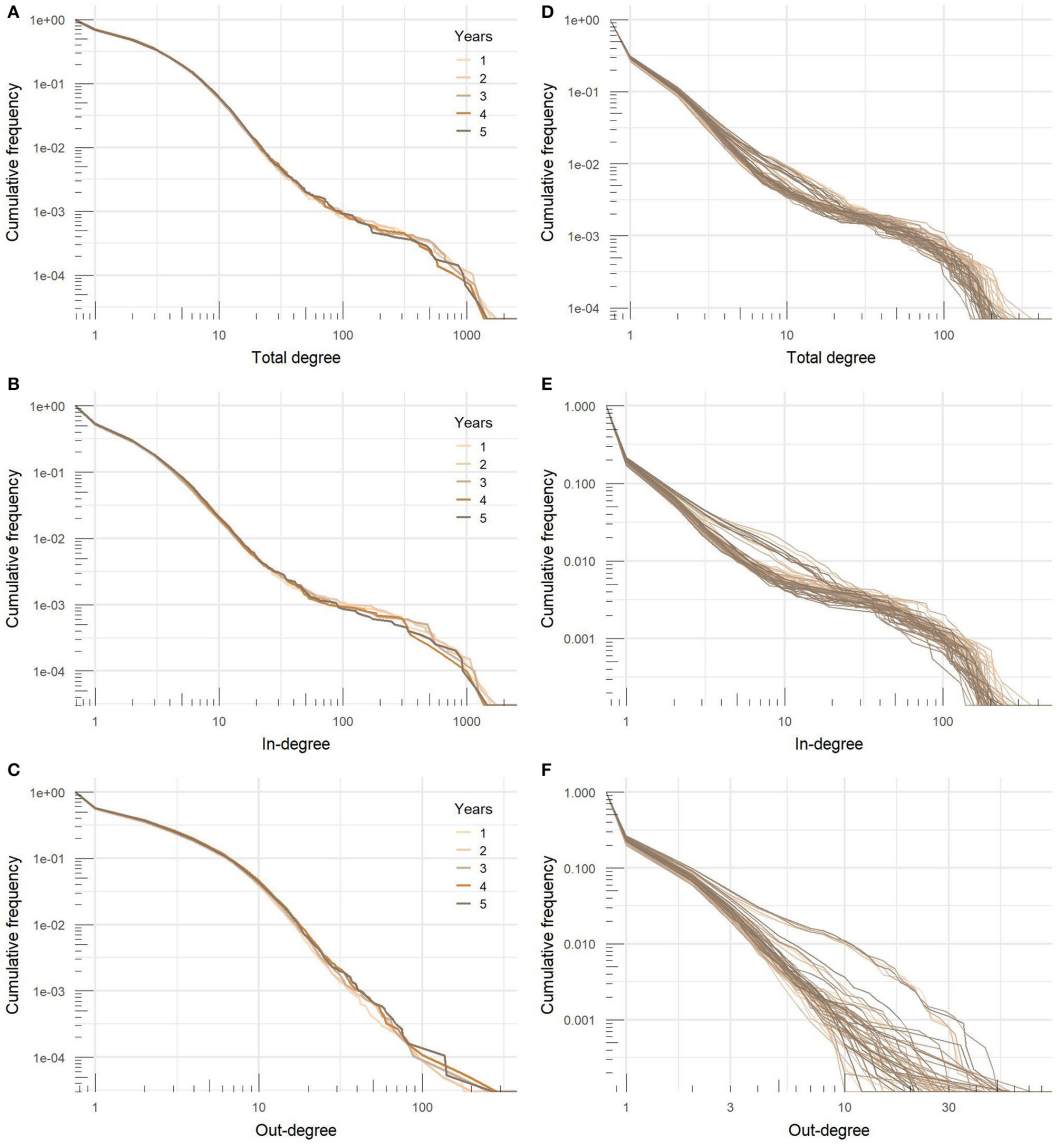
Degree distributions of **(A)** yearly total degree, **(B)** in-degree, and **(C)** out-degree and **(D)** monthly total degree, **(E)** in-degree, and **(F)** out-degree of cattle movement networks.

Figure 5 shows cumulative distributions of the total, in- and out-degree in yearly and monthly networks on a logarithmic scale. Distributions were heavy-tailed with more or less apparent bimodal structure, which is a consequence of a large proportion of nodes with relatively low degree values and a small number of nodes with extremely high values of degree centrality. In the yearly networks, ten percent of nodes with the highest total degree centrality accounted for ~54% of all movements, top 20% of nodes for 70% of movements, and top 30% of nodes for 80% of movements. The variation between years was very low and the range was covered with less than a 1.2 percentage point difference.

**Figure 6:**
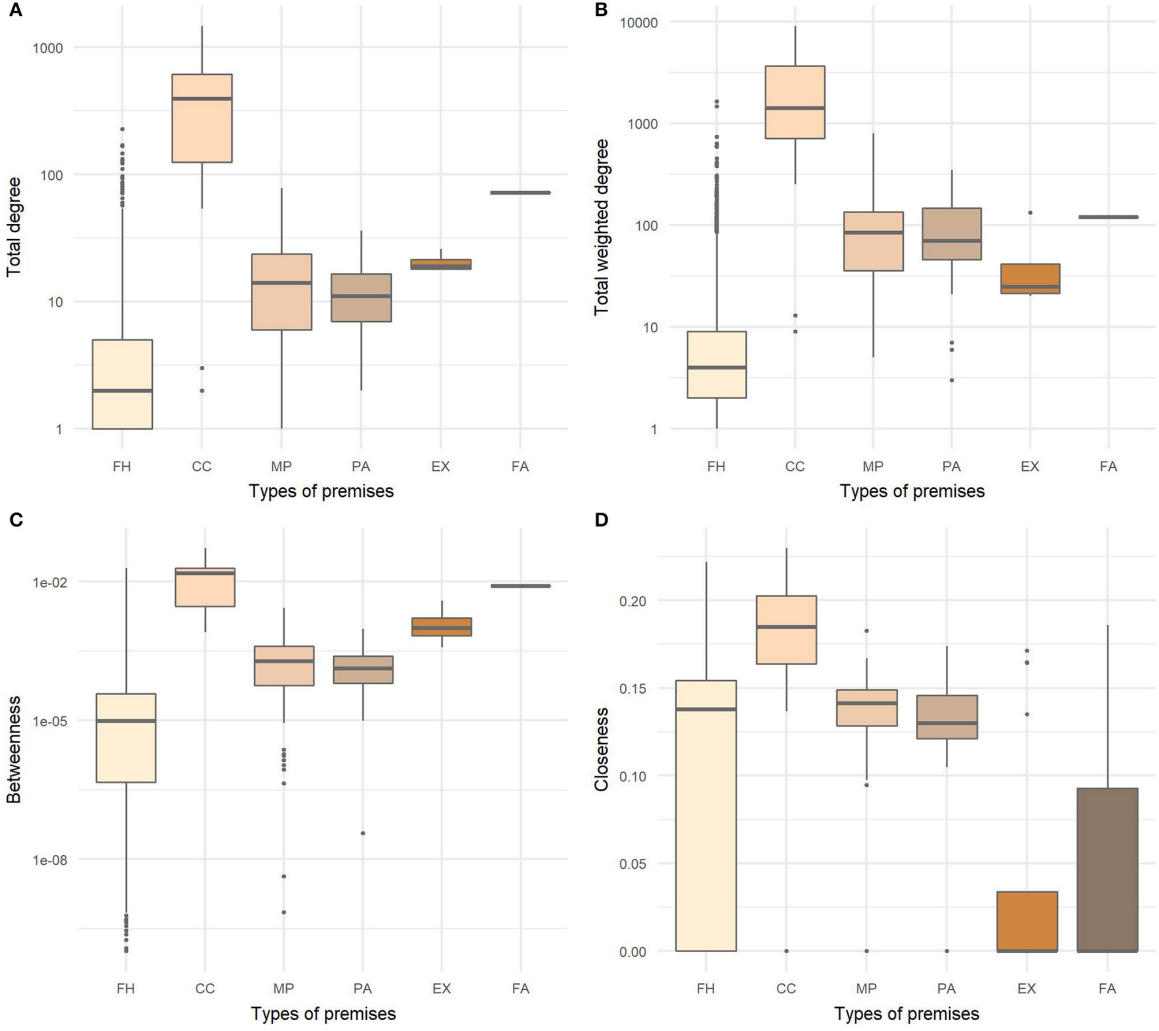
Distributions of **(A)** total degree, **(B)** total weighted degree, **(C)** betweenness, and **(D)** closeness centralities by type of premises in the last year of studied cattle movement network. FH, farm holdings; CC, collection centers; MP, mountain pastures; PA, pastures; EX, exhibitions; FA, fairs.

For all node level measures, there were statistically significant differences between types of premises (Figure 6). Mountain pastures significantly differed from farm holdings and exhibitions in all measures as well as exhibitions from pastures. No significant difference was found between fairs in relation to farm holdings and exhibitions. Collection centers were shown to be the most influential type of premises in the global networks since they had the highest values of all observed measures.

Compared to the cattle trade networks, the random networks with the same number of nodes and arcs exhibited less structuredness and more homogeneous connectedness. The average shortest path length and diameter were higher than observed in the trade networks. The clustering coefficient was one to two-folds lower. At the same time, the giant components included many more nodes than our studied networks; GWCC 95% and GSCC 40% of all nodes.

**Figure 7:**
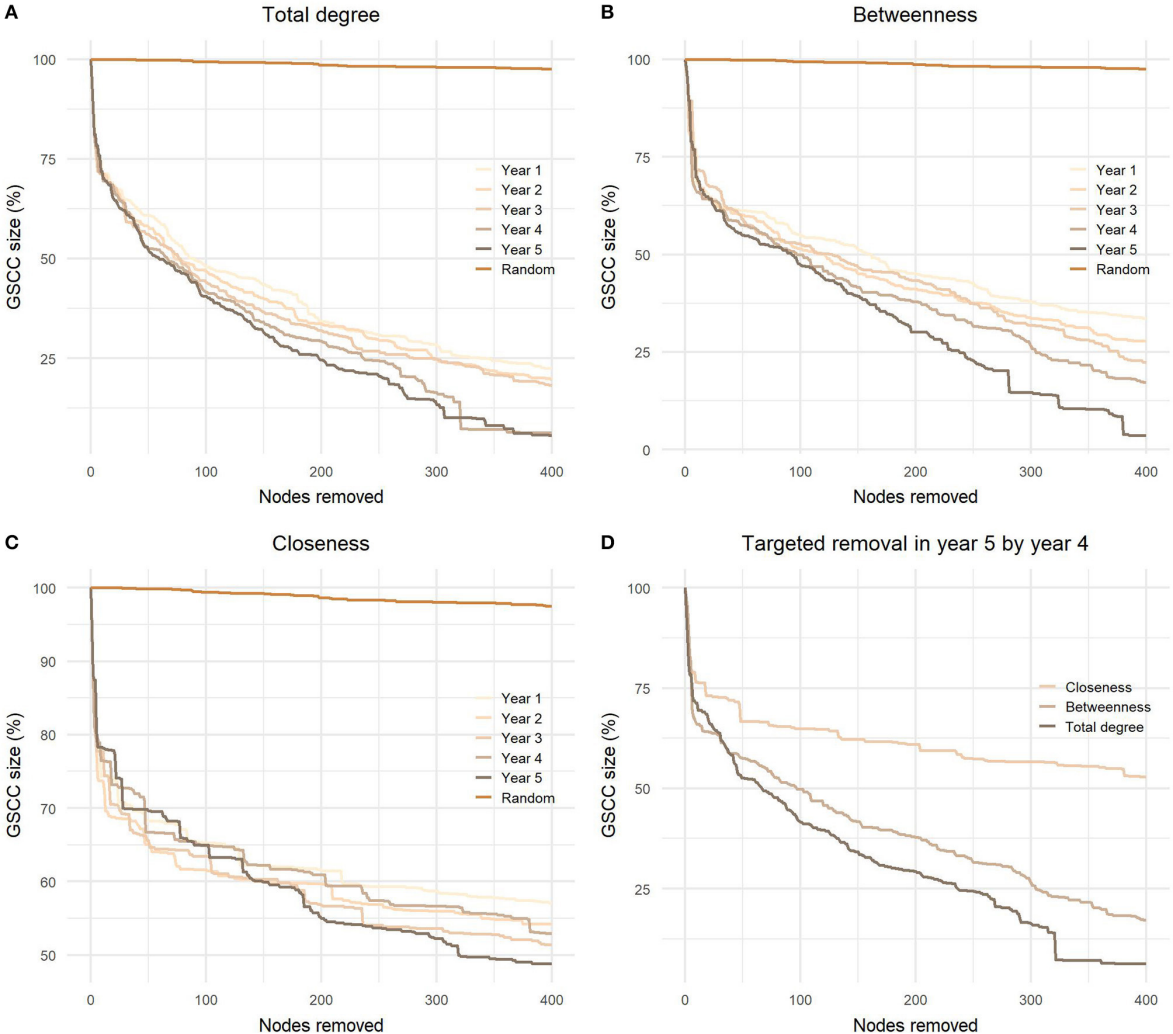
Targeted node removal in the fifth yearly network based on **(A)** total degree, **(B)** betweenness, and **(C)** closeness centrality from the first to the last studied year and **(D)** comparison of effectiveness between these three centralities in the fourth yearly network on disintegration of the GSCC in the fifth yearly network. GSCC, giant strongly connected component.

As demonstrated in Figure 7, the most successful strategy to reduce the size of GSCC was node removal based on the total degree centrality in the fifth year, hence in the same year as the removal was performed. When we removed 0.1% of all nodes in the network, the results were quite similar for degree and betweenness centrality; the mean sizes of the remaining GSCCs were 59.7 and 60.3%, respectively, and the variation between years was low. Closeness centrality was less efficient and the mean size of remaining GSCC was 69.3% after removing 0.1% of nodes and 53.7% after removing 1% of all nodes. The difference between degree and betweenness centrality became apparent when we removed 0.5% nodes, the sizes of remaining GSCCs ranged from 26.0 to 38.5% and 32.9 to 45.8%, respectively. It also became apparent that using the latest data gives better results. After removing 1% of nodes based on the values for the fourth year, the GSCC size dropped to 18.4% when using ordering based on betweenness and 6.5% when using the total degree.

### SI Model of Possible Paratuberculosis Spread on the Temporal Network

**Figure 8:**
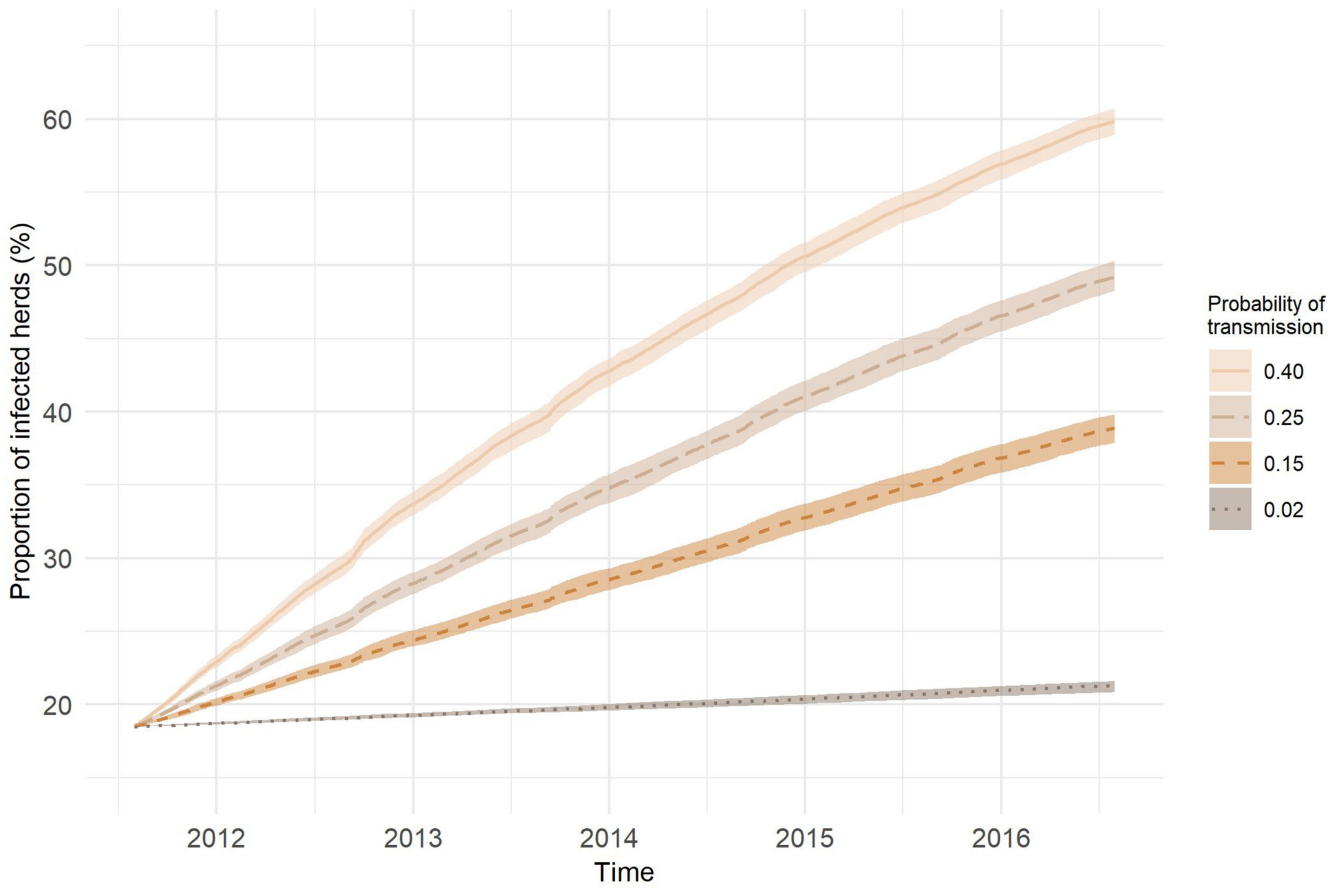
SI infection curves (lines—average over 1,000 realizations; area—range) in the temporal network representation of cattle movements for different probabilities of transmission.

**Figure 9:**
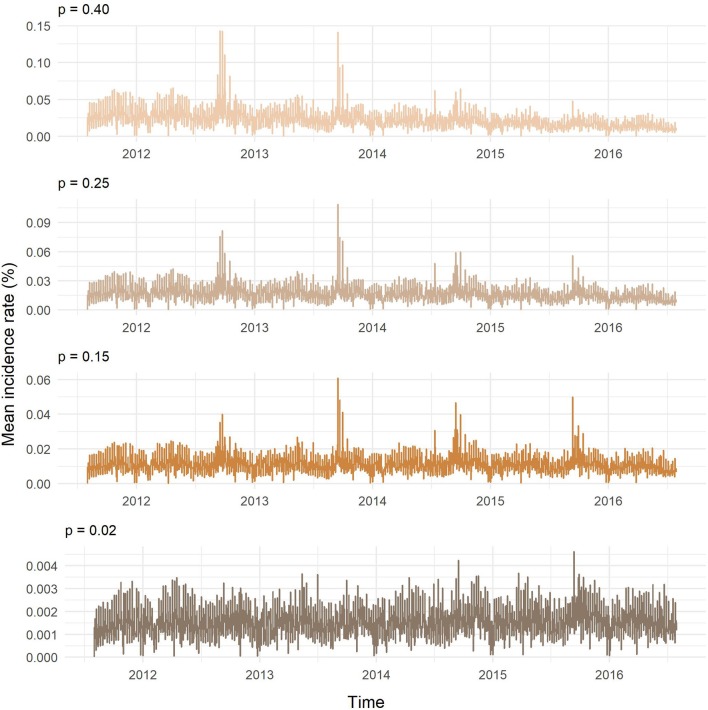
Mean daily incidence rate of newly infected farm holdings for different probabilities of transmission (p).

Figure 8 presents the results of the SI model on the temporal network predictions of possible MAP spread between cattle herds in Slovenia. The probability of transmission largely affected the proportion of farms being infected and the velocity of spread on the network. At the end of the study period, the predicted proportion of newly infected herds obtained with one thousand realizations for our chosen transmission probabilities of 0.02, 0.15, 0.25, and 0.40 were: 2.81, 20.40, 30.72, and 41.36%, respectively. As expected, the higher was the probability of transmission the steeper were the slopes of infection curves. Even though we started the simulation with randomly selected initially infected farm holdings and one hundred different diluted networks, the variations in the proportion of infected herds for each transmission probability were low. Standard deviation was increasing with time and reached maximum toward the end of the study period, except for the transmission probability of 0.40, where the maximum standard deviation was observed at day 1,073. Maximum standard deviations from the lowest to the highest probability of transmission were: 0.01, 0.31, 0.33, and 0.32%. The average number of newly infected farm holdings per day were: 0.57, 4.12, 6.20, and 8.34, respectively (Figure 9). Four distinct peaks in the incidence rate can be observed, concentrated mainly in September and October. In none of the scenarios, the infection reached a saturation; however, in the estimated and worst-case scenario, the velocity of disease spread decreased toward the end of the study period.

## Discussion

The first purpose of this study was to explore the Slovenian cattle trade network characteristics to determine if network analysis could provide support in targeted paratuberculosis surveillance and control measures. The second aim was to evaluate possible MAP spread on the studied network. We focused on paratuberculosis because it causes large economic losses and consequently puts pressure on one of the most important parts of the Slovenian agricultural sector.

A static representation of the network was used for the characterization of global and local network structure with special attention given to the epidemiological implications. By definition, the static network is an approximation of the temporal network, since it does not include the information about the time of movements and hence does not take into account the succession of movements, which is an integral part for understanding a disease dynamic. Therefore, it overestimates the network connectedness and the outbreak size and does not provide any information about the outbreak duration (36, 42). Even though there are considerable shortcomings of using static network analysis in veterinary epidemiology, it is deemed sufficient when trying to provide an overview of the network topology. Based on the current knowledge in comparison to temporal network analysis, the static analysis offers clear definitions of global and local network measures that can be fairly easily computed without the use of programming languages. For example, large animal trade networks can be analyzed with the Pajek software, which can be used by non-computer experts. In fact, we used Pajek for the whole static network analysis except for the generation of random networks and the targeted node removal. Another important benefit of using static network analysis is that the results are easily interpretable and tangible, which render them useful for planning targeted intervention measures.

To determine whether network analysis could offer support in increasing the efficiency of paratuberculosis intervention measures in Slovenia, we used yearly snapshots of our cattle trade network to quantify year to year consistency of cattle movements and heterogeneity in the importance of premises. Monthly networks were used to assess seasonality. We chose these aggregation windows because it was previously argued that networks aggregated over longer time periods may adequately address chronic or slowly spreading diseases (56). Even though the Slovenian cattle movement network is in general smaller and less active compared to other European cattle trade networks, it shares a number of properties with them, such as similar degree distributions, average shortest path length, and diameter (40, 41, 51, 52, 57).

In the monthly networks, two peaks in the frequency of animal movements were apparent during late spring to early summer and in autumn, which is due to the grazing period. Similar seasonal patterns were described also in other countries with grazing practices (40, 51, 52). In our cattle movement networks, this seasonal pattern was reflected in all monthly global network measures except diameter and was consistent through time. In addition, global and local network measures in the yearly networks showed consistency throughout the studied period. As in studies of cattle movements in other countries (27, 37, 40), node level measures have also proven to be of great assistance in identifying premises that are disproportionately important from the epidemiological perspective compared to other premises in the Slovenian cattle trade network. Collection centers without exception were among these premises. The finding that this type of premises has central positions within the network and consequently poses a higher risk for disease spread is consistent with results from other studies (41, 52). As expected, pastures and mountain pastures were also shown to be important for disease spread. This is substantiated with the circumstances of the grazing system since animals stay at the designated location for a longer period and can homogeneously mix with all other animals at the same location, both of which do not hold for collection centers, exhibitions or fairs. Since MAP can be shed in feces in high quantity and the main route of infection is a fecal-oral route, pastures provide ideal conditions for disease spread.

Even though the network measures were consistent over time, year to year contacts between premises turned out to be preserved only to some extent. The same conclusion was reached by Marquetoux et al. (37) with the evaluation of the consistency of contacts between pairs of farms. We have observed this in three instances: First, with the calculation of yearly changes in premises affiliation to components of the bow-tie partition (Figure 4). Second, with correlations in network measures between different years. And third, by targeted node removal (Figure 7). The latter was used to test whether the local network measures could increase the efficiency of intervention measures. We simulated a risk-based selection of premises in the last studied year based on premises ranking in the previous years and tracked changes in network connectedness when individual premises were removed. The results showed that disassociation of the network was substantially improved when the removal of the nodes was informed and the more recent the information have been the better were the results. Konschake et al. (58) showed that nodes identified as the most important in a static network remained relevant also in the temporal network approach.

For the evaluation of a possible disease spread through cattle movements, we used the full temporal resolution of the network. This was modeled in terms of a temporal network, where the causal accessibility is captured and consequently, we obtained a more realistic representation of possible disease dynamics. The SI model presented herein shows possible MAP spread based on the empirically estimated true prevalence of paratuberculosis in Slovenia and four scenarios for different transmission probabilities. These simulations allowed for an estimation of the probability distributions of paratuberculosis prevalence at any time during the studied period. Nevertheless, the objective was not to get the exact prediction of the paratuberculosis prevalence, but rather to study the implications of network structure for the MAP spread. Given the low infection rate and the slow course of the disease, within-herd dynamics should be included. At least herd demographic data and the probability of infection on the animal level should be implemented when using the temporal network model for a reliable prediction of disease prevalence. Therefore, our model overestimates the disease spread and represents a worst-case outcome.

Based on the results from the static network analysis and the relatively high proportion of initially infected herds, the resulting high prevalences at the end of the study period were expected. The within-herd prevalence and the probability of moved animals being infected had a significant influence on the velocity of disease spread through the network. The peaks in daily incidence rates of newly infected herds were observed at the end of the grazing period. Since the peaks were not present in the first autumn of any scenario, in the best-case scenario and at the end of the worst-case scenario, the threshold in the number of infected premises of certain type appeared to exist. These crucial premises were farm holdings with common pastures. At the beginning of the simulation, the threshold was not reached because the initially infected premises were randomly selected. In the worst-case scenario, the disease reached the majority of these premises within the first 3 years.

Our model did not include the possibility of the infection fading out, but this was regarded as a reasonable assumption since the extinction of paratuberculosis at the population level without the implementation of effective control measures is unlikely to occur (26). We are aware that this is an oversimplified model, but we are focusing on the phase of spreading where more complex mechanisms, such as countermeasures or recovery, are not yet established since the disease has a slow onset and there are no control measures in place in Slovenia. In addition, studies suggest an increase in the prevalence of paratuberculosis in most countries with a significant dairy industry (1). The high increase in paratuberculosis prevalence is supported by a number of recent studies. Whittington et al. (59) reported that from 48 countries included in the survey approximately half had more than 20% of herds infected. In some developed countries (e.g., Italy, France, Germany, United Kingdom) prevalence exceeded 40%. Even though the reported prevalences were high the authors warned that under-reporting and under-estimations of paratuberculosis prevalence are common.

The model included a couple of underlying assumptions that have probably contributed to an overestimation of the transmission potential. We did not distinguish between animals based on sex, age or breed and thus, the risk each animal posed for disease transmission was assumed the same. We did not consider the time an animal spent at individual premises. Consequently, if for example an infected animal was introduced to the farm holding and the next day, the same holding sold an animal, the probability that it was infected was the same as for animals sold months after the introduction of the infected animal in the herd. It was assumed that all animals at the same premises have equal chance to get infected, which for example excludes the possibility that animals at the farm holding of different categories were housed in different buildings or that animals at the exhibition were effectively separated. Another limitation is that we did not include import or export movements and regarded our network as a closed system. Imports were not considered since we only had the data on the country from which the animal was imported and thus the model would assign the same health state to the whole country.

On the other hand, we adopted some assumptions that probably led to an underestimation of the potential disease transmission. The period in which the study of paratuberculosis prevalence was conducted and the starting date of our network are 2.5 years apart. Therefore, the proportion of initially infected herds was probably higher than the one we used in the model. As the prevalence in this study was calculated, we did not have data on which herds were infected and therefore used random sampling, which may affect the result. With the exclusion of end nodes from the network, we adopted the assumption that animals moved to slaughterhouses, dead animals or their parts do not pose any risk for the transmission of disease. This may not be the case for MAP, but the spread through these routes is perceived to be unlikely. Furthermore, we inherently neglected other potential transmission pathways; for example, movement of other susceptible or non-susceptible animal species, movement of owners, workers or veterinarians, transmission via vehicles, shared equipment or bioaerosol (7, 12). The main reason being the lack of empirical data and the fact that tackling direct transmission through cattle movements should be the first step in efforts to control MAP spread between herds.

In this article, we presented the first in-depth analysis of the Slovenian cattle trade characterized as a complex network. We argue that network analysis may aid in the optimization of paratuberculosis surveillance and control in Slovenia. When providing advice, the latest available data should be used. The results of this study are applicable for risk-based interventions for other cattle diseases that spread mainly through animal movements. Even though the SI model was based on several simplifying assumptions, it offered an insight into the importance of between-farm movements for disease propagation in the cattle population. Moreover, the employed approach with diluted networks may also be used to model other between-herd transmission pathways.

## Data Availability Statement

The datasets generated for this study will not be made publicly available because they contain detailed information on individual holdings. The data analyzed in this study was obtained from the Administration of the Republic of Slovenia for Food Safety, Veterinary Sector and Plant Protection (AFSVSPP). Requests to access these datasets should be directed to the AFSVSPP, gp.uvhvvr@gov.si.

## Author Contributions

All authors participated in the conception of the study. TK performed the network analysis and drafted the manuscript. TK and HL prepared the SI model. All authors contributed to the interpretation of the results, wrote sections of the manuscript, reviewed, and approved the submitted version.

### Conflict of Interest

The authors declare that the research was conducted in the absence of any commercial or financial relationships that could be construed as a potential conflict of interest.

## Footnotes

^1^http://pisrs.si/Pis.web/pregledPredpisa?id=PRAV7492

^2^http://pisrs.si/Pis.web/pregledPredpisa?id=PRAV11848

^3^https://www.stat.si/StatWeb/en/Field/Index/11

^4^https://github.com/hartmutlentz/TemporalNetworkAccessibility

^5^https://pxweb.stat.si/SiStatDb/pxweb/en/30_Okolje/
